# Topical Ozone Therapy—A Novel Modality in the Treatment of a Complicated Wound after Knee Joint Ligament Operation as a Consequence of Traffic Accident—Case Report

**DOI:** 10.3390/medicina58091259

**Published:** 2022-09-12

**Authors:** Jarosław Pasek, Tomasz Pasek, Sebastian Szajkowski, Grzegorz Cieślar

**Affiliations:** 1 Faculty of Health Sciences, Jan Długosz University in Częstochowa, 13/15 Armii Krajowej St., 42-200 Częstochowa, Poland; 2 Rehabilitation Unit of the St. Barbara Provincial Specialist Hospital No.5 in Sosnowiec, Medyków Square 1, 41-221 Sosnowiec, Poland; 3 Faculty of Medical Sciences, Medical University of Mazovia in Warsaw, 8 Rydygiera St., 01-793 Warszawa, Poland; 4 Department of Internal Medicine, Angiology and Physical Medicine, Faculty of Medical Sciences in Zabrze, Medical University of Silesia in Katowice, 15 Stefana Batorego St., 41-902 Bytom, Poland

**Keywords:** physical treatment, ozone therapy, postoperative chronic wound, complication after traffic accident

## Abstract

Background and objectives: For many years, medicine has been looking for effective methods to be used in the treatment of chronic wounds. Pharmacological treatment is insufficient and does not give expected results of treatment. In the comprehensive treatment of wounds, physical medicine methods have been used, which are characterized by high efficiency and safety as well as relatively low costs of the therapy. Efficient application of a novel therapeutic modality in the form of topical ozone therapy in the treatment of a difficult-to-heal wound of the left knee joint after surgery due to the rupture of the anterior cruciate ligament and damage to the medial meniscus because of a previous road accident in a 61-year-old female patient is presented. Methods: Topical ozone therapy treatment in the form of the “Ozone bag” with the use of an oxygen-ozone mixture (2.86% ozone and 97.14% of oxygen) with a concentration of 40 µg/mL was applied to the wound area. The therapeutic cycle consisted of two series of 10 treatment sessions lasting 20 min each, performed every day for 5 days a week, and carried out for 6 weeks. Results: Topical ozone therapy caused complete healing of the complicated wound remaining after orthopaedic surgery, which allowed the patient to live independently without experiencing pain, to move without elbow crutches, and to perform daily activities independently and ultimately to return to work.

## 1. Introduction

The most common consequences of road accidents are multiple injuries, most often head injuries and fractures of limbs. Limb injuries are the second leading cause of hospitalization for traumatic reasons and in many cases require surgery. The consequence of each surgical procedure is a wound, which in most cases heals on its own by rapid obliteration. Some wounds do not heal properly for various reasons. In damaged tissues, pathological vasospasms, slower blood flow, oedema, and inflammation, as well as the accumulation of exudates, the contents of which are toxic to wounds, may occur. The existing postoperative wound healing disorders may lead to the development of infection, dehiscence of the wound edges and the formation of abnormal scar tissue. They can also cause life-threatening systemic complications. Due to the above factors, the postoperative wound healing process may take a long time [1,2].

Until now, medicine has not had a single golden mean that can be effectively used in the treatment of difficult-to-heal wounds. According to current recommendations, properly conducted treatment of such wounds requires an interdisciplinary approach and should include local treatment combined with pharmacotherapy, compression therapy, the use of specialized dressings and physiotherapy procedures. In some cases, surgery is also necessary, especially in the case of traumatic wounds. The inclusion of physical medicine procedures as part of the comprehensive treatment plan significantly accelerates the treatment process and alleviates the accompanying pain [3].

For several years, an increasing number of studies has been carried out to determine the effectiveness of ozone therapy used in the treatment of wounds, especially those that are difficult-to-heal and are complicated. Ozone (O_3_), which is an active form of oxygen, is most often used in the form of dry baths in an oxygen-ozone mixture in the proportion of 2.86% by volume of ozone and 97.14% by volume of oxygen, applied as the so-called “Ozone bag”. The recommended therapeutic ozone concentration is 50–120 μg ozone/mL oxygen. In ozone generators producing ozone from oxygen supplied from a cylinder, the ozone concentration at the device outlet is 5–70 µg/mL, and the maximum pressure is 0.06 MPa [4,5].

Ozone applied to the wound area has a very strong bactericidal effect, whose mechanism of action prevents the development of resistance to bacterial strains. Apart from bactericidal activity, ozone also reduces the severity of inflammation by inhibiting the migration of mast cells, reducing the release of lysosomal enzymes and some acute phase proteins, and stimulating the formation of eosinophils as well as antioxidants. In erythrocytes, in turn it causes an increase of the negative charge, resulting in the erythrocytes becoming more flexible. The process of their rolling is inhibited and their passage through the narrowed capillaries because of the disease process is facilitated. Ozone also activates the Krebs cycle by increasing the production of 2,3-diphosphoroglycerate and the intensity of glycolysis in erythrocytes, which leads to an increase in the amount of oxygen and ATP released to the tissues and the intensification of cytochrome C oxidation. Thanks to these effects, tissue oxygenation and nutrient supply are increased [4,5,6].

To date, most papers in the available literature present the beneficial therapeutic effects of topical ozone therapy in the treatment of hard-to-heal wounds, as well as venous leg ulcers or diabetic foot ulcers, and only a few assess the efficacy of this method in case of post-traumatic or postsurgical wounds [6].

Therefore, the aim of this case report was to present the high efficacy of this novel therapeutic modality, regarding acceleration of wound healing and reduction of accompanying pain, in the treatment of a difficult-to-heal wound of the left knee joint after surgery due to rupture of the anterior cruciate ligament and damage to the medial meniscus resulting from a previous road accident in a 61-year-old female patient.

## 2. Case Report

A 61-year-old female patient came to the Department of Internal Medicine, Angiology and Physical Medicine of Specialistic Hospital no. 2 in Bytom due to a dehiscence of a left knee surgical wound after a traffic accident (the patient was hit by a car at a pedestrian crossing). Immediately after the accident, the patient, as an emergency case, was taken to the orthopaedics ward of the Hospital Emergency Unit. The physical examination and imaging examinations (X-ray and MRI of the knee joint) performed in the orthopaedics ward revealed dislocation, sprain, and tear of the anterior cruciate ligament, as well as damage to the medial meniscus of the left knee joint of the 3rd degree (according to O’Donoghue triad), that was confirmed in intra-surgery observation. On the day of admission to the orthopaedic ward, the patient underwent an open surgical procedure consisting of suturing the ligaments with Ti Screw ES 5.0 BIOMET anchors and suturing the medial meniscus. The course of the surgical procedure and post-operative period were without complications. After surgical procedure, moving about with the use of crutches and not leading of the operated lower extremity were prescribed. The patient was supplied with a knee functional orthesis (0–15° in extension) and performed active and isometric exercises of the operated lower extremity. The patient was discharged with the recommendation of continuing the above rehabilitation and continued medication, consisting of: Clexane 0.4 mL 1 × 1 ampoule s.c., Cyclo 3 fort 2 × 1 tablet and Poltram Combo in tablets in case of pain intensification. After 14 days, the postoperative sutures were removed in Orthopaedic Consulting Unit, and after a further few days, the separation of wound lips occurred. The patient returned to the orthopaedic department, where the consulting orthopaedic physician proposed a revision procedure due to the separated wound, but the patient did not give consent to this form of treatment without any specific reason.

Then the patient was admitted to the emergency unit at the Clinical Department of Internal Medicine, Angiology and Physical Medicine for a consultation regarding the selection of methods of continued treatment of the complicated wound. The consulting physician, after taking the patient’s history and physical examination as well as analysis of the provided medical documentation, qualified the patient for conservative treatment of the wound to be performed in the form of a series of topical ozone therapy procedures that were planned to be performed at Physical Medicine Ward, which is a part of the Clinical Department, on outpatient basis.

The interview and medical documentation showed that the patient had been suffering from arterial hypertension for 6 years (measurement value on admission to the Clinic-145/90 mmHg) and hypercholesterolemia (total cholesterol concentration-220 mg/dL). The patient denied the use of stimulants, except for the occasional consumption of small amounts of alcohol. The patient’s height was 1.67 m and body mass 79 kg (BMI was 28 kg/m^2^).

Before starting the series of physical procedures, the patient assessed the intensity of wound pain experienced during the preceding week at the average value of 4/5 points in 10-point Visual Analogue Scale (VAS) (in which 0 points corresponds to lack of pain and 10 points correspond to absence of pain and 10 points correspond to the most intensive pain experienced in patient’s life), that was indicative of a moderate pain. She also subjectively assessed the quality of life by means of the EuroQol scale (in which 100 points correspond to the best imaginable quality of life and 0 points correspond to the worst imaginable quality of life), scoring 30 points.

**Figure 1:**
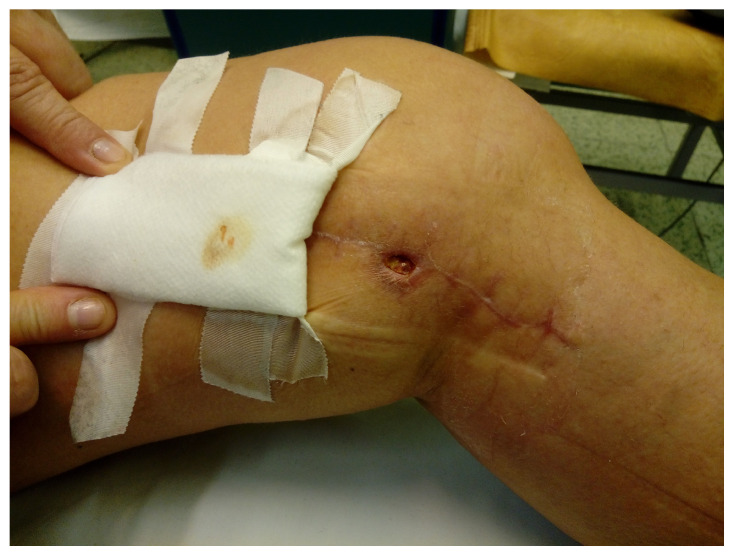
The local state of wound before the topical ozone therapy procedures.

The physical examination of wound performed before the beginning of the series of the topical ozone therapy procedures showed the separation of the lips of the wound was localized on the medial surface of the knee in its proximal part (length ca. 1 cm) with accompanying singes of inflammation in the tissues surrounding the wound (Figure 1).

## 3. Topical Ozone Therapy Procedures

The patient underwent topical ozone therapy with the use of the Ato-3 device (Metrum Cryoflex, Blizne Łaszczyńskiego, Poland) with the application of a gaseous oxygen-ozone mixture (2.86% ozone and 97.14% oxygen) with the concentration of 40 µg/mL applied to the wound area in the form of the so-called “Ozone bag”. The entire therapeutic cycle consisted of two series of 10 procedures lasting 20 min each, performed daily for 5 days a week, excluding Saturdays and Sundays. The interval between consecutive series was 2 weeks. After each procedure, a nanocrystalline sterile Acticoat Flex 3 dressing with silver was applied to the wound to aid the healing process and to maintain the wound moisture content. In addition, to mechanically protect the wound, an Allevyn Life dressing was applied to the wound, thanks to which the exudate was drained to the absorbent layer, and its high absorption capacity prevented the contents from percolating or returning to the wound bed. In topical antiseptic treatment, Octenisept was used to wash the wound.

During the topical ozone therapy, the patient continued previously prescribed physical exercises and, due to the separation of the wound lips, further pressure relief of operated lower extremity was provided.

Moreover, the patient continued the current pharmacological treatment for arterial hypertension (Prestarium and Cozaar) and hypercholesterolemia (Simvacard).

During the cycle of topical ozone therapy procedures, after its completion, and two months after the end of the treatment, the progress of the wound healing was documented by means of photographs. Moreover, after the completion of the cycle, the assessment of wound pain intensity with the use of the VAS scale as well as the assessment of the quality of life with the use of EuroQoL were performed.

## 4. Results

**Figure 2:**
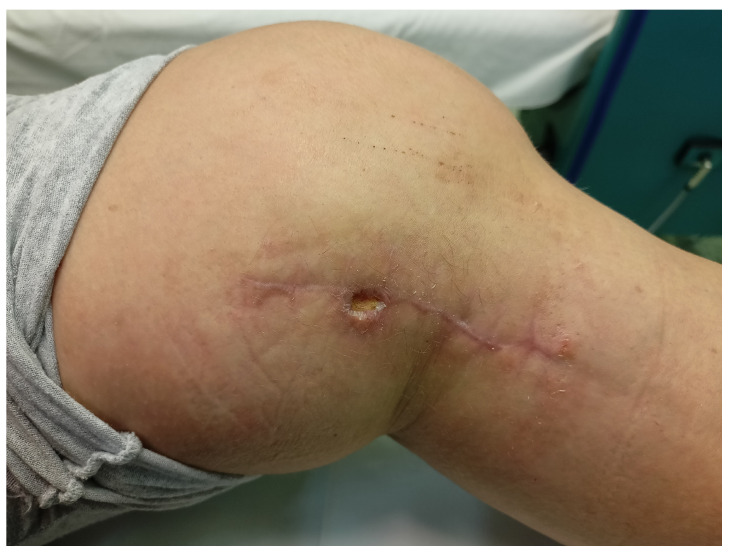
The local state of the wound after 10 topical ozone therapy procedures.

**Figure 3:**
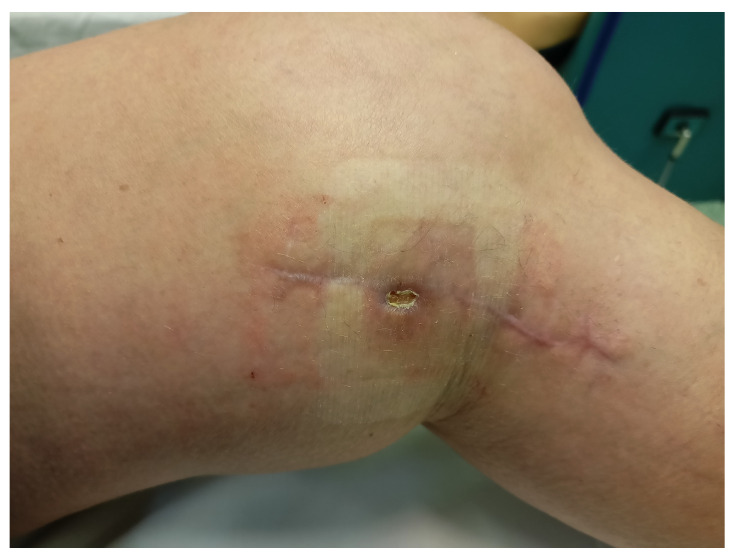
The local state of the wound after 15 topical ozone therapy procedures.

The applied cycle of topical ozone therapy procedures resulted in the gradual cleansing of the wound and the formation of new granulation tissue, which created conditions for the skin allowing persistent islets of the epidermis to extend out from the wound edges and from the bottom of the wound, and consequently led to the complete healing of the wound. The intensity of the inflammatory reaction as well as skin congestion and temperature in the treated area were also significantly reduced (Figure 2 and Figure 3).

**Figure 4:**
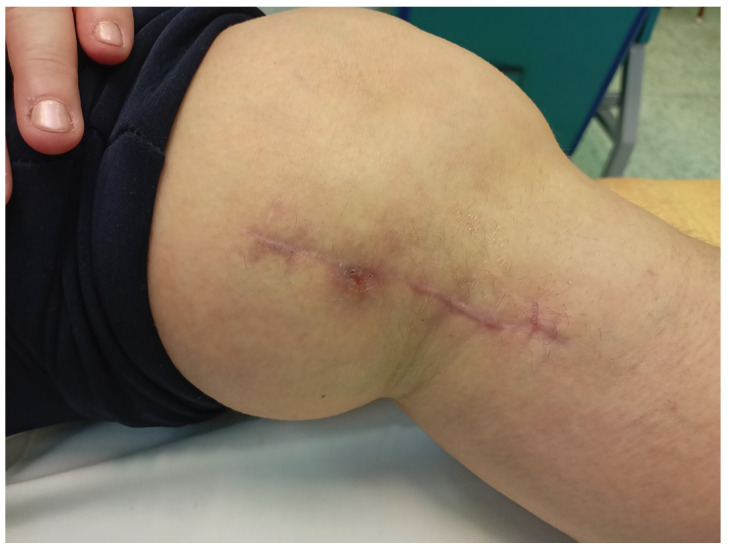
The local state of the wound after the end of topical ozone therapy procedures (total of 20 procedures within 6 weeks from the start of treatment).

The final treatment result is presented in Figure 4.

At the end of the 6-week therapeutic cycle, the pain previously reported by the patient was completely relieved (0 points in the VAS scale), while the quality of life determined in the subjective EuroQol scale was assessed by the patient at 90 points.

**Figure 5:**
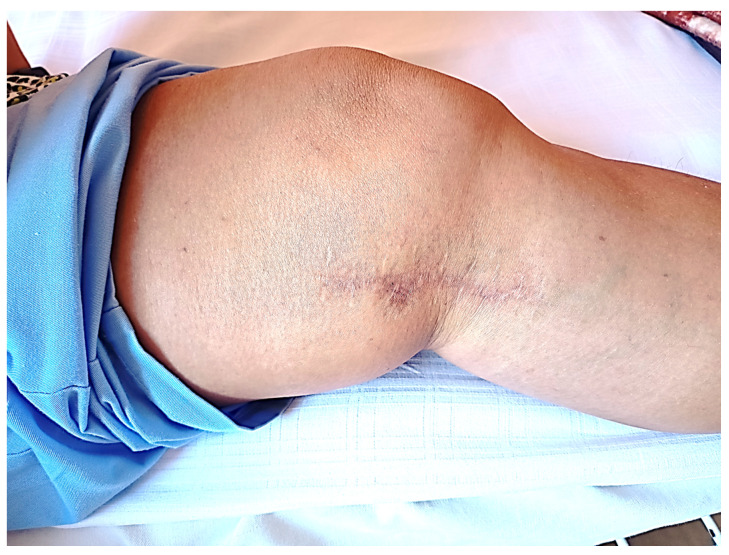
The local state of the wound 2 months after completing the topical ozone therapy procedures.

During treatment, the patient did not report any side-effects of the therapy. Two months after the end of treatment, complete wound healing was observed during a follow-up visit at the Physical Medicine Ward (Figure 5).

## 5. Discussion

In the postoperative period, the application of antiseptics, proper wound care and the use of appropriate dressings play a key role. In the case of abnormal wound healing, which is one of the postoperative complications, it is important to promptly diagnose and, if necessary, implement appropriate treatment, which, as our case has shown, should also include physical medicine procedures [7,8].

The World Health Organization (WHO) reports that the number of postoperative complications, including nosocomial infections, in European countries ranges from 5 to 10% of all hospitalized people. This means that as many as 100 million people suffer from them annually, including—as the analysis conducted by the European Legal Center shows—almost 600,000 patients in Poland [9].

The most common postoperative complications related to wound healing disorders include infection, wound dehiscence, and abnormal scarring. In the case of wound dehiscence, where it affects the superficial layers of the body’s integuments, such as skin or subcutaneous tissue, surgical debridement is usually an effective treatment. This complication is especially dangerous when wound dehiscence affects deep tissues. In such cases, it seems necessary to perform revision surgery in the operating theatre and to reunite the wound [1,10].

In patients with difficult-to-heal wounds, oxygen-ozone therapy may be instrumental in accelerating of healing and in reducing the intensity of pain due to its disinfecting properties and in stimulating the ability to remove endogenous free oxygen radicals from the tissues surrounding the wound [5,11,12].

Considering that the wound dehiscence in the presented case concerned both superficial tissues and deeper ones, in the absence of the patient’s consent for repeated surgical debridement, the decision was made to include in a comprehensive therapy a topical application of ozone. The aim of this was to relieve the pain and to stimulate the tissue regeneration process by improving oxygenation and the quality of local blood supply to the tissues undergoing treatment. An important element of the comprehensive therapy was also the use of appropriate dressings for the wound, which ensured proper care of it (sterility and mechanical protection).

For Degli Agosti et al., who used this method in a 46-year-old patient after amputation of the right tibia and fibula resulting from a motorcycle accident, the results were equally favourable, as in the presented case. Two months after the injury, in their case the wound did not heal despite prior treatment and the use of appropriate dressings, and the patient complained of persistent pain in the wound area. After adding a 5-week cycle of ozone therapy, the wound was completely healed [6].

Fitzpatrick E. et al. conducted a literature search regarding the potential benefits of ozone therapy in case of chronic wounds. They assessed the research available in the following databases: Google Scholar, PubMed, and the Cochrane Library. Nine of the studies reviewed, presenting the results of treatment for a total of 453 patients, met the inclusion criteria and were used in a meta-analysis, which showed that ozone therapy used as part of comprehensive therapeutic management improved healing and accelerated the treatment of chronic wounds, when compared to classic therapy [13].

In turn, Anzolin et al. analysed 28 articles on the topical application of ozonated oil in acute and chronic inflammations of soft tissues complicating the wound healing process. The results of the analysed studies confirmed the high effectiveness of ozonated oil use in the case of disturbances in the healing of complicated skin wounds resulting from the reduction of microbial infection, stimulation of the wound cleansing process, modification of the inflammatory phase of wound healing, and stimulation of angiogenesis processes and the biological and enzymatic reactions that favour the improvement of oxygen metabolism, improving the quality of the resulting scar. In addition to supporting the wound healing process, ozonated oil also alleviates symptoms associated with skin burns, prevents skin discoloration after injuries and alleviates pain resulting from aphthous ulcers [14].

To sum up, postoperative wounds that are difficult-to-heal, which are a complication of various metabolic disorders related to insufficient supply of oxygen and nutrients to cells, are the basis for the developing infection, which leads to the formation of necrosis in nearby tissues. An untreated or improperly treated postoperative wound may also penetrate deep into the integuments, creating ulceration which results in its dehiscence. These complications extend the initially expected hospitalization time, significantly increase the cost of treatment, and often also bring about persistent adverse cosmetic effects. For the above reasons, proper treatment of postoperative wounds is currently one of the priorities for most surgical treatment disciplines [1,15,16].

The presented case proves that local ozone therapy can be a valuable element of comprehensive treatment of difficult-to-heal postoperative wounds, which will potentially enable the optimal implementation of the above-mentioned priority.

## 6. Conclusions

Adjuvant topical ozone therapy procedures were applied to the presented patient after the dehiscence of the wound resulting from knee joint surgery due to traumatic damage to the anterior cruciate ligament and the medial meniscus inflicted because of a road accident. We confirmed the high efficacy of this novel therapeutic modality in the treatment of a postsurgical complicated wound in the form of complete healing and reduction of the intensity of inflammation and complete subsidence of the accompanying pain.

## Data Availability

The datasets used and/or analysed during the current study are available from the corresponding author on reasonable request.
